# Overweight in Adolescence and Young Adulthood in Association With Adult Cerebrovascular Disease: The NFBC1966 Study

**DOI:** 10.1161/STROKEAHA.123.045444

**Published:** 2024-06-06

**Authors:** Ursula Mikkola, Ina Rissanen, Milja Kivelä, Harri Rusanen, Eero Kajantie, Jouko Miettunen, Markus Paananen

**Affiliations:** Research Unit of Population Health (U.M., I.R., M.K., J.M., M.P.), University of Oulu, Finland.; Clinical Medicine Research Unit (E.K.), University of Oulu, Finland.; Medical Research Center Oulu, Oulu University Hospital and University of Oulu, Finland (U.M., I.R., M.K., H.R., E.K., J.M.).; Julius Center for Health Sciences and Primary Care, University Medical Center Utrecht, the Netherlands (I.R.).; Department of Neurology, Oulu University Hospital, Finland (H.R.).; Population Health Unit, Finnish Institute for Health and Welfare, Helsinki, Finland (E.K.).; Department of Clinical and Molecular Medicine, Norwegian University of Science and Technology, Trondheim, Norway (E.K.).; Social and Health Care Services, Western Uusimaa Wellbeing Services County, Helsinki, Finland (M.P.).

**Keywords:** adolescent, birth cohort, body mass index, cerebrovascular disorders, overweight, young adult

## Abstract

**BACKGROUND:**

Risk factors for cerebrovascular disease in adulthood are well known. However, research on individuals’ risk factors throughout their life span has been limited. This prospective cohort study aims to determine the effect of body mass index (BMI) and its changes in adolescence and young adulthood on early onset cerebrovascular disease.

**METHODS:**

This study includes 10 491 people (5185 women) from the Northern Finland Birth Cohort 1966. Height, weight, and BMI were measured at ages 14 and 31 years. Sex- and age-specific BMI ranges were used to define overweight and obesity. Data on ischemic and hemorrhagic cerebrovascular diseases between ages 14 and 54 years were extracted from national hospital and death registers. Cox proportion hazard models (95% CI) were used to estimate associations between BMI or its changes and cerebrovascular disease, while adjusting for sex, smoking, educational level, BMI at the other time point, and age at menarche for women. Additionally, sex-BMI interactions were calculated.

**RESULTS:**

A total of 452 individuals (4.7%) experienced cerebrovascular disease during the follow-up. The risk of ischemic cerebrovascular disease was increased for overweight women at ages 14 years (hazard ratio [HR], 2.49 [95% CI, 1.44–4.31]) and 31 years (HR, 2.13 [95% CI, 1.14–3.97]), as well as for obese women at ages 14 years (HR, 1.87 [95% CI, 0.76–4.58) and 31 years (HR, 2.67 [95% CI, 1.26–5.65]), with normal weight as the reference. These results were independent of earlier or later BMI. Similar associations were not found among men. The risk of hemorrhagic cerebrovascular disease was increased at age 31 years both among obese women (HR, 3.49 [95% CI, 1.13–10.7) and obese men (HR, 5.75 [95% CI, 1.43–23.1). The risk of any cerebrovascular disease related to overweight at age 14 years was 2.09× higher among girls than boys (95% CI, 1.06–4.15). The risk of ischemic cerebrovascular disease related to obesity at age 31 years was 6.96× higher among women than men (95% CI, 1.36–35.7).

**CONCLUSIONS:**

Among women, being overweight in adolescence or young adulthood increases the risk of cerebrovascular disease, especially ischemic, independent of their earlier or later BMI.

**See related article, p 1866**

Cerebrovascular disease is the second-leading cause of death and the third-leading cause of death and disability combined worldwide.^1^ While most cerebrovascular diseases occur in the elderly population, the incidence of cerebrovascular diseases in the young has increased by 23% in 1 decade.^2^ The potential inability of young survivors to return to work after experiencing cerebrovascular disease can have a substantial socioeconomic impact.^3,4^ Primary prevention remains the most effective approach for reducing the burden of cerebrovascular disease. Modifiable risk factors, such as obesity and smoking, are the main targets of primary prevention.^5^

Obesity is a well-known risk factor for cerebrovascular disease,^6,7^ and childhood obesity has been associated with an increased risk of cerebrovascular disease later in life.^8^ However, most studies have focused on body mass index (BMI) at 1 time point and have not considered its fluctuations throughout life. Childhood obesity has been shown to predict adulthood obesity.^9^ In Finland, 18% of girls and 29% of boys aged 2 to 16 years are overweight,^10^ and 35% of women and 47% of men aged 18 to 29 years are overweight.^11^ Such a high prevalence poses a likely threat to public health in terms of cerebrovascular disease.

In the Northern Finland Birth Cohort 1966, previous research has established that low early childhood weight and height increase the risk of ischemic cerebrovascular disease in adulthood among women, and the timing of weight gain during childhood plays a significant role in the development of cerebrovascular disease risk among women.^12^ Sex disparity in cerebrovascular disease occurrence has been well identified. The age-adjusted incidence of cerebrovascular disease is higher in men than in women, except in the elderly, which is mostly a result of longer life expectancies in women.^13^ The hypothesis of this study was that BMI in adolescence or young adulthood displays some sex disparity.

In this study, we used the Northern Finland Birth Cohort 1966 to study the participants’ BMI at ages 14 and 31 years in association with the risk of early adulthood cerebrovascular disease (<55 years). Additionally, we investigated whether changes in BMI between these ages or sexes modify the risk. Our aim was to assess the age- and sex-specific relationship between BMI and total, ischemic, and hemorrhagic cerebrovascular diseases.

## METHODS

### Data Availability Statement

The Northern Finland Birth Cohort 1966 data are available from the University of Oulu, Infrastructure for Population Studies.^14^ Permission to use the data can be applied for research purposes via the material request portal (www.oulu.fi/nfbc). The European Union general data protection regulation (679/2016) and the Finnish Data Protection Act are followed. The use of personal data is based on the written informed consent of the cohort participants, which may cause limitations to its use.

### Design of Study and Sample

The Northern Finland Birth Cohort 1966 is a prospective, general population-based birth cohort containing data on 12 055 pregnant women and their 12 058 children born alive in the provinces of Oulu and Lapland with an expected date of birth in 1966.^15^ Data collection was initiated antenatally, and the cohort has been regularly followed up ever since. The collected data are linked to existing, comprehensive nationwide registers.

Excluded from the study were participants whose informed consent was not obtained (n=133) and those who had a cerebrovascular disease before the age of 14 (n=5) years. This study includes 9608 participants at age 14 years and 8294 participants at age 31 years (Figure). The follow-up continued until their first cerebrovascular disease, death, moving abroad, or the end of 2020, whichever came first.

**Figure.**
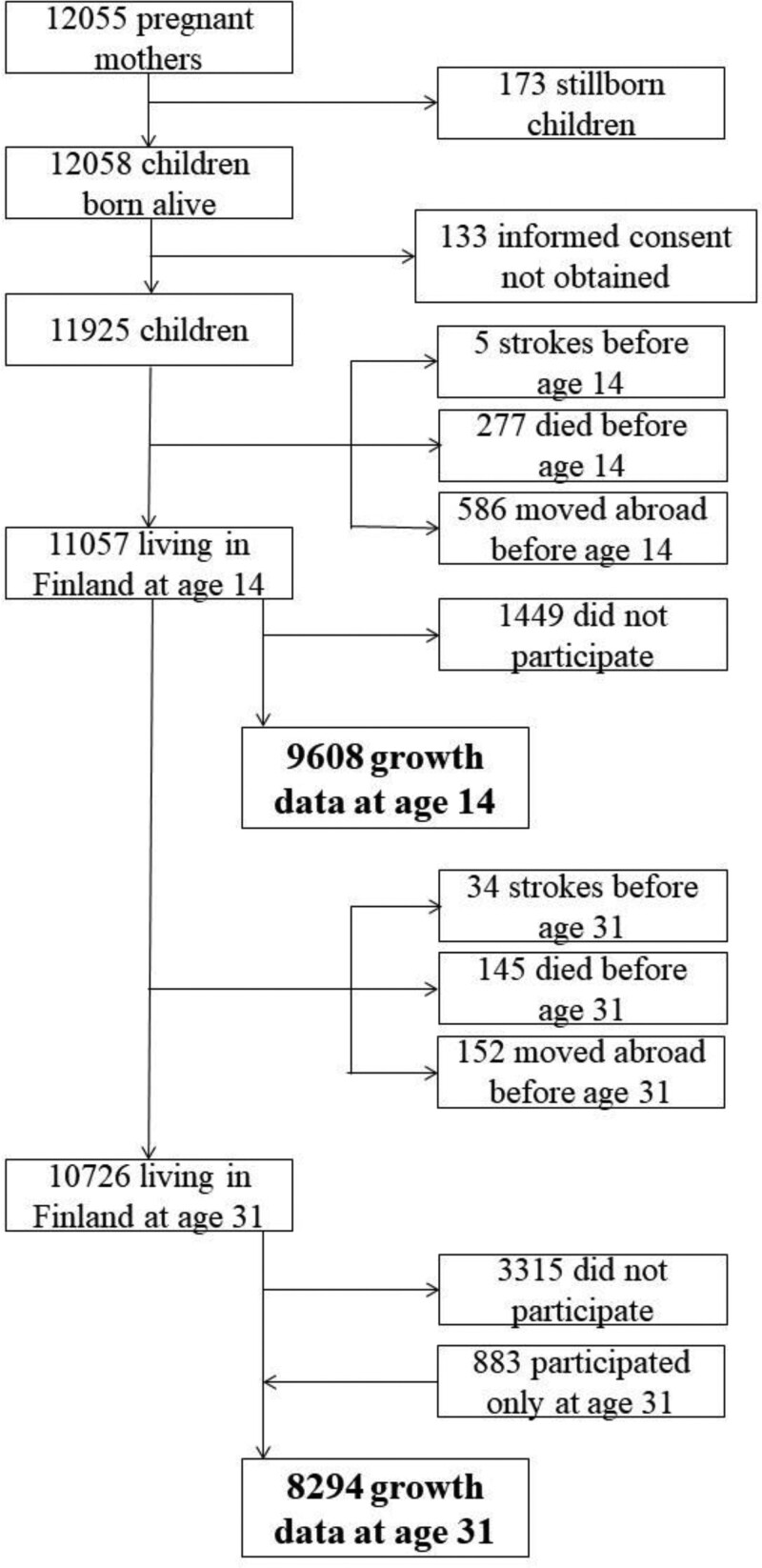
Study population flow chart.

At age 14 years, the follow-up was performed using a postal questionnaire that included questions about growth and general health. The questionnaire was first sent to the children, then to their parents if the children did not respond, and ultimately to the regional school offices and school health nurses if neither the children nor their parents responded.

At age 31 years, the follow-up involved a postal questionnaire and a clinical health examination. The questionnaire included questions about participants’ growth, general health, and living situation. The clinical health examination was conducted for cohort members living in Northern Finland and the Finnish capital area and performed by nurses in local health centers.

Permission to gather register data was obtained from the Ministry of Social Affairs and Health, and the study was approved by the Ethical Committee of Northern Ostrobothnia Hospital District in Oulu, Finland. Informed consent was obtained from all the participants.

This study was performed and reported in accordance with the Strengthening the Reporting of Observational Studies in Epidemiology checklist.

### Growth Data

The current sample included 10 491 participants with growth data either at age 14 or 31 years (Figure). At age 14 years, BMI data were collected through a postal questionnaire from 9608 participants, with 50.1% boys and 49.9% girls. At age 31 years, BMI data were obtained either through a postal questionnaire or clinical examination from 8294 participants, with 48.4% men and 51.6% women. We have primarily used the measured values (n=5916), and if not available, then the self-reported values were used (n=2378). Most participants (n=7411) reported BMI or were measured at both ages.

BMI was calculated as weight in kilograms divided by height in meters squared (kg/m^2^). Height and weight were measured at the exactitude of 0.1 cm or 0.1 kg. Age- and sex-specific BMI ranges defined overweight and obesity in both ages. At age 14 years, categories were defined separately for girls and boys in accordance with the World Health Organization guidelines: underweight (BMI ≤5th percentile), overweight (BMI 85th–94th percentile), and obesity (BMI ≥95th percentile).^16,17^ For girls, underweight was defined as BMI <16 kg/m^2^, normal weight as 16≤BMI<23 kg/m^2^, overweight as 23≤BMI<25.5 kg/m^2^, and obesity as BMI ≥25.5 kg/m^2^. For boys, underweight was defined as BMI <16 kg/m^2^, normal weight as 16≤BMI<22 kg/m^2^, overweight as 22≤BMI<24.3 kg/m^2^, and obesity as BMI ≥24.3 kg/m^2^. At age 31 years, the body mass categories were defined as follows: underweight as BMI <20 kg/m^2^, normal weight as 20≤BMI<25 kg/m^2^, overweight as 25≤BMI<30 kg/m^2^, and obesity as BMI ≥30 kg/m^2^. Normal weight was chosen as the reference category.

An additional analysis was conducted to study the effect of waist circumference on stroke, as abdominal body fat has proven to be a stronger predictor of stroke risk than BMI.^18^ We created a binary variable of either a high-risk or low-risk waist circumference. At the age of 31 years, the high-risk cutoff for waist circumference was 94 cm for men and 80 cm for women.

### Cerebrovascular Disease

The outcome variable was cerebrovascular disease. Strokes and transient ischemic attacks were identified from the nationwide Care Register for Health Care and Causes of Death Register based on medical records.^19^ The diagnostic coding has been based on the World Health Organization *International Classification of Diseases* (*ICD*) in Finland since 1967. Cerebrovascular diseases were classified as follows: subarachnoid hemorrhages (*ICD*-8 430; *ICD*-9 430; *ICD*-10 I60 and I69.0), intracerebral hemorrhages (*ICD*-8 431; *ICD*-9 431; *ICD*-10 I61 and I69.1), ischemic strokes (*ICD*-8 432–434; *ICD*-9 433–434; *ICD*-10 I63 and I69.3), transient ischemic attacks (*ICD*-8 435; *ICD*-9 435; *ICD*-10 G45), and other cerebrovascular diseases (*ICD*-8 436–438; *ICD*-9 436–437; *ICD*-10 I64–I68, I69.4, and I69.8). Stroke syndromes (*ICD*-9 438; *ICD*-10 G46) were classified according to etiological subcodes (*ICD*-9 430–437; *ICD*-10 I60–I67) or as other cerebrovascular diseases if subcodes were not present. The linkage to other data with pseudonymization was fully complete for cerebrovascular disease diagnoses. Ischemic strokes and transient ischemic attacks were defined as ischemic cerebrovascular diseases, and subarachnoid hemorrhages and intracerebral hemorrhages were defined as hemorrhagic cerebrovascular diseases. For analyses of any cerebrovascular diseases, ischemic strokes, hemorrhagic strokes, transient ischemic attacks, and other cerebrovascular diseases were combined. Traumatic subarachnoid hemorrhage or intracerebral hemorrhage, epidural hematoma, or subdural hematoma were not considered cerebrovascular diseases. Participants with ≥2 diagnoses were classified according to their primary diagnosis of the first event.

### Covariates

At age 14 years, covariates were smoking, parents’ socioeconomic status, and age at menarche for girls. Smoking status was categorized as either smoker (twice per week or more) or nonsmoker.

Socioeconomic status was defined as high if a person had a full-time working father or mother. First, we used the father’s occupational status, but if the father was not working full-time, then socioeconomic status was defined based on the mother’s occupational status. If no information was obtained from the father’s side, then the mother’s occupational status was used.

For girls, the age at menarche was classified into 3 groups: 9 to 11 years for early onset, 12 to 14 years for average onset, and 15 to 18 years for late onset. The average onset age served as the reference category. The age at menarche was asked retrospectively in a postal questionnaire at age 31 years.

At age 31 years, covariates were smoking pack-years and the participant’s own educational level. Smoking pack-years were estimated by multiplying self-reported smoking years with the number of packs of cigarettes smoked per day. This measure was chosen because it is estimated to predict accumulated cerebrovascular disease risk better than current smoking status at the time.^20^

We created a binary variable of either low or high educational level. Postsecondary education, vocational school, vocational training course, or no occupational education were classified as having a low educational level, and polytechnic or university degrees were classified as having a high educational level.

### Statistical Analyses

Cox proportional hazards models were used to estimate the associations of BMI at ages 14 and 31 years in relation to cerebrovascular disease during follow-up. All models were stratified by sex. The follow-up times were calculated separately from ages 14 and 31 years onward. Results are presented with hazard ratios (HRs) and their 95% CIs. Adjustments at age 14 years were smoking, parents’ socioeconomic status, and age at menarche for women. Adjustments at age 31 years were smoking pack-years and the participant’s own socioeconomic status. Sensitivity analyses were conducted, repeating the analyses without transient ischemic attacks (Tables S1 and S2). The effect of change in BMI was analyzed by adjusting the results at age 14 years with BMI at age 31 years, and vice versa.

The sex-BMI interactions were analyzed with Cox proportional hazard model for each BMI category. At age 14 years, the interaction model was adjusted with smoking, parents’ socioeconomic status, and the sex interactions of those. At age 31 years, the interaction model was adjusted with smoking and one’s own socioeconomic status.

E-values were calculated to assess the potential contribution of unmeasured confounding factors for each of the models (Tables S3 through S8).

All analyses were performed using IBM SPSS Statistics 28.0 for Windows (IBM Corp).

## RESULTS

### Characteristics of Sample

The length of follow-up was 372 675 person years from age 14 years onward and 189 093 person years from age 31 years onward. The mean follow-up time per participant was 38.8 (SD, 6.1) years from age 14 years onward and 22.8 (SD, 3.1) years from age 31 years onward. During follow-up, a total of 452 (4.7%) participants had a cerebrovascular disease. Of them, 141 (31.2%) were ischemic strokes, 180 (39.8%) transient ischemic attacks, 51 (11.3%) subarachnoid hemorrhages, 32 (7.1%) intracerebral hemorrhages, and 48 (10.6%) other cerebrovascular disease events. The median age at onset was 47.1 (SD, 8.5) years for ischemic stroke, 48.9 (SD 5.3) years for transient ischemic attack, 45.5 (SD 13.5) years for intracerebral hemorrhage, and 43.8 (SD 10.6) years for subarachnoid hemorrhage.

Growth characteristics are shown in Table 1. At age 14 years, the mean BMI for girls was 19.4 kg/m^2^ (SD, 2.5) and for boys 19.3 kg/m^2^ (SD, 2.6). At age 31 years, the mean BMI for women was 24.0 kg/m^2^ (SD, 4.5) and for men 25.3 kg/m^2^ (SD, 3.6).

**Table 1.**
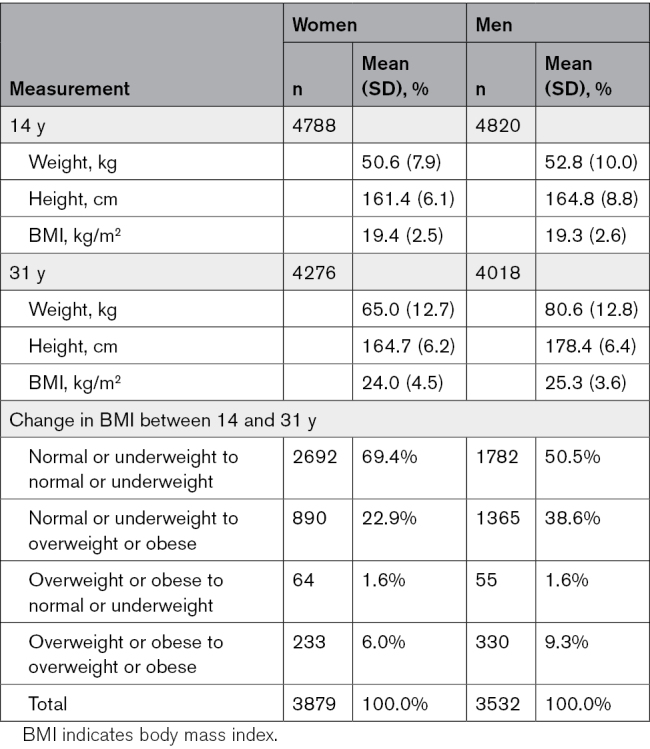
BMI Measurements in Adolescence and Young Adulthood

### Adolescence BMI and Cerebrovascular Disease

At age 14 years, overweight was associated with an increased risk for cerebrovascular disease (HR, 2.49 [95% CI, 1.55–4.00]) and especially ischemic cerebrovascular disease (HR, 2.49 [95% CI, 1.44–4.31]) later in life among women (Table 2). Despite a change in BMI by age 31 years, being overweight at age 14 years was still associated with an increased risk of cerebrovascular disease among women, especially ischemic cerebrovascular disease (Table 3). Similar associations were not found among men (Table 4).

**Table 2.**
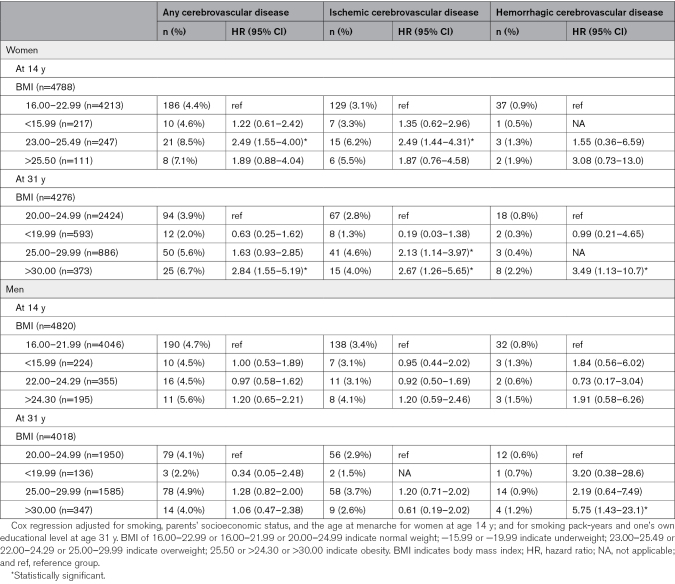
Associations of BMI in Adolescence and Young and Cerebrovascular Event Under the Age of 55 Years Stratified by Sex

**Table 3.**
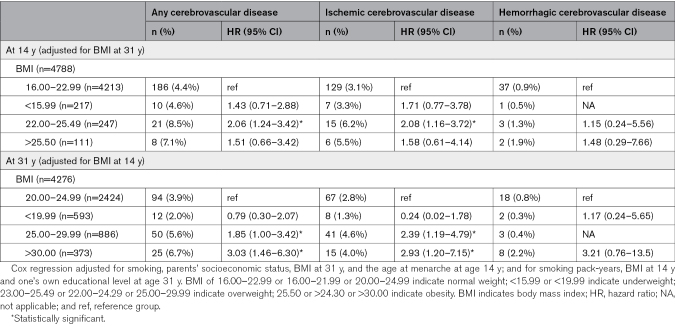
Associations of Change in BMI Between the Ages 14 and 31 Years and Cerebrovascular Event Under the Age of 55 Years Among Women

**Table 4.**
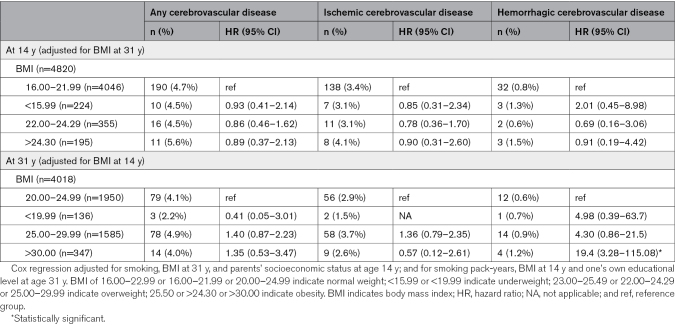
Associations of Change in BMI Between the Ages 14 and 31 Years and Cerebrovascular Event Under the Age of 55 Years Among Men

The sex-BMI interaction of any cerebrovascular disease related to overweight was 2.09× higher among women than men (95% CI, 1.06–4.15) and the HR of ischemic cerebrovascular disease related to overweight was 2.29× higher among women than men (95% CI, 1.01–5.19; Table 5).

**Table 5.**
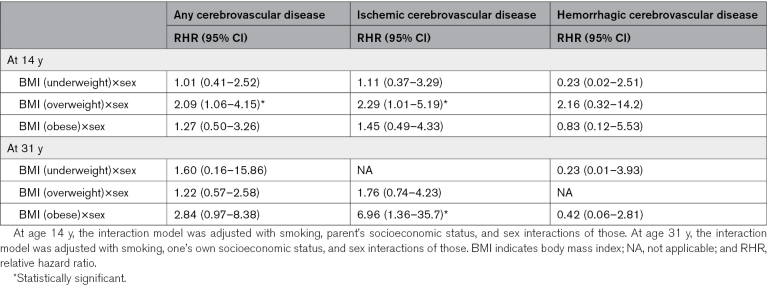
Interaction Term of BMI and Sex and Cerebrovascular Event Under the Age of 55 Years in Women Compared With Men

Sensitivity analyses were conducted, repeating the analyses without transient ischemic attacks, and the results were not affected (Tables S1 and S2).

### Adulthood BMI and Cerebrovascular Disease

At age 31 years, being overweight was associated with an increased risk of ischemic cerebrovascular disease among women (HR, 2.13 [95% CI, 1.14–3.97]; Table 2). Also, among women, obesity (HR, 2.84 [95% CI, 1.55–5.19]) increased the risk of any cerebrovascular disease in later life and was associated with an increased risk of both ischemic cerebrovascular diseases, with an HR of 2.67 (95% CI, 1.26–5.65) and hemorrhagic cerebrovascular diseases, with an HR of 3.49 (95% CI, 1.13–10.7; Table 2). Among men, obesity (HR, 5.75 [95% CI, 1.43–23.1]) was associated with an increased risk of especially hemorrhagic cerebrovascular disease later in life (Table 2). No other associations were found among men. Regardless of BMI at age 14 years, being overweight or obese at age 31 years was still associated with an increased risk of cerebrovascular disease among women, particularly ischemic cerebrovascular disease (Table 4). Additionally, having a high-risk waist circumference was associated with an increased risk of cerebrovascular disease among women (HR, 3.22 [95% CI, 1.85–5.61]) but not among men.

The sex-BMI interaction of ischemic cerebrovascular disease related to obesity was 6.69 times higher among women than men (95% CI, 1.36–35.7; Table 5).

Sensitivity analyses were conducted, repeating the analyses without transient ischemic attacks, and the results were not affected (Tables S1 and S2).

## DISCUSSION

We have examined how adolescent and young adult BMI are associated with early onset cerebrovascular disease. Women who were overweight at ages 14 or 31 years had an increased risk of cerebrovascular disease. Both associations were independent of BMI at the other time point (14-year BMI independent of 31 years, and vice versa), suggesting that overweight carries a risk throughout adolescence and young adulthood, at least for women. The association between high BMI and cerebrovascular disease was considerably stronger for women than for men, among whom we found limited evidence of associations between adolescent or young adult BMI and early onset cerebrovascular disease. The E-values (Tables S3 through S8) are low to moderate, implying that little unmeasured confounding would be needed to explain away an effect estimate. In the main findings, the E-values are moderate. The possibility of important, unmeasured confounding should be considered when interpreting our results. Additionally, having a high-risk waist circumference was associated with an increased risk of cerebrovascular disease only in women, supporting our other findings.

We found out that overweight is a risk factor at ages 14 and 31 years for women, irrespective of BMI at the other age. Our results suggest that being overweight at age 14 years was associated with later cerebrovascular disease risk, despite having lost weight by age 31 years. Furthermore, being overweight at age 31 years was associated with later cerebrovascular disease risk, despite having a normal weight at age 14 years. The current literature supports this finding. Studies have found that weight gain during puberty and adolescence is associated with a risk of adult cerebrovascular disease.^21,22^ Another study found that weight gain during early adult life, but not mid-life, is associated with an increased risk of cerebrovascular disease.^23^ In contrast, a study in Helsinki, Finland, found no association between BMI at age 11 years and adult cerebrovascular disease, but cerebrovascular disease was predicted by a lower BMI earlier in childhood.^24^ Taken together, these findings highlight the importance of maintaining a healthy weight throughout life.

Several studies have identified the association between overweight and an increased risk of cerebrovascular disease in young adults.^6,7^ Although this is a well-known risk factor, the pathophysiological mechanisms underlying this link remain controversial.^25^ One of the suggested pathways is the chronic inflammation state caused by excess fatty tissue, which, in turn, leads to difficulty in blood flow and an increased risk of blockage.^25,26^ Overweight is also associated with several secondary risk factors for cerebrovascular disease, such as high blood pressure and diabetes.^25^

In previous studies, overweight and obesity have been associated with a higher incidence of total and ischemic cerebrovascular disease in both women and men.^27,28^ Interestingly, another study has reported a significant association between high BMI and the incidence of ischemic cerebrovascular disease only in men.^29^ One study found that underweight, overweight, and obesity were associated with a higher hemorrhagic cerebrovascular disease incidence in men but not in women.^27^ However, participants in the aforementioned studies were middle-aged and older at the onset of cerebrovascular disease, whereas we studied the effect of BMI earlier in life and early onset cerebrovascular disease.

There are some potential explanations for our findings since cerebrovascular disease risk factors unique to women have previously been recognized.^30^ Mechanisms explaining the stronger association of overweight with cerebrovascular disease in women could be due to differences in sex hormones and pubertal timing between women and men.^30^ Research on the role of endogenous estrogen levels in women is relatively limited, but in a Danish prospective cohort study, neither high nor low estrogen levels were associated with an increased risk of ischemic cerebrovascular disease.^31^ In some previous studies, earlier age at menarche has been associated with greater cardiovascular disease risk,^32–34^ but not in all studies.^35,36^ It is also known that overweight women have an earlier age at menarche,^37^ but the causal pathways possibly linking age at menarche, overweight, and cerebrovascular disease remain poorly known. Standardizing the analyses for age at menarche among women did not affect the results in the present study.

This study has limitations. First, cerebrovascular diseases in early adulthood are quite rare,^2^ and despite having a large population-based cohort with extensive follow-up data, only 452 cerebrovascular diseases were recorded. Second, the common problem with BMI is that it does not measure body composition or fat distribution^38^; however, the use of BMI is still commonly accepted.^39^ Third, BMI was based on self- or parent-reported heights and weights for all cohort members at age 14 years and for 29% of them at age 31 years. Fourth, we did not have complete BMI data during adolescence and young adulthood. Finally, the study population was quite homogeneous and entirely Finnish, so there is limited generalizability of the findings.

The present study has several strengths, the main ones being the prospective method of data collection, the length of follow-up, and the large size of the cohort population. We performed separate analyses for both sexes and analyzed sex-BMI interactions. Due to having data at 2 different time points, we were able to assess the effect of changes in BMI. There were no significant amounts of missing data. Additionally, we conducted sensitivity analyses by eliminating transient ischemic attacks, yet the results remained the same. Information on cerebrovascular disease diagnoses was reliable and complete, sourced from nationwide registers.^19^

## CONCLUSIONS

Overweight at ages 14 and 31 years is independently associated with adult cerebrovascular diseases among women but not among men. The association between childhood overweight and adult cerebrovascular disease is independent of overweight or obesity in adulthood, highlighting the importance for children to achieve and maintain healthy weights. The mechanisms underlying these sex-specific associations remain to be elucidated.

## ARTICLE INFORMATION

### Acknowledgments

The authors thank all cohort members who participated in the study and the researchers of the Northern Finland Birth Cohort project center.

### Sources of Funding

The Northern Finland Birth Cohort 1966 (NFBC1966) received financial support from University of Oulu [65354, 24000692]; Oulu University Hospital [2/97, 8/97, 24301140]; Ministry of Health and Social Affairs [23/251/97, 160/97, 190/97]; and the National Institute for Health and Welfare [54121]; and the European Regional Development Fund [539/2010 A31592]. This study was supported by Orion Research Foundation, Päivikki and Sakari Sohlberg Foundation, and Paulo Foundation. The funders had no role in study design, data collection and analysis, decision to publish, or preparation of the manuscript.

### Disclosures

Dr Rissanen reports employment by Duodecim Publishing Company Ltd. Dr Kajantie reports grants from Academy of Finland. The other authors report no conflicts.

### Supplemental Material

Tables S1–S8

## Supplementary Material



## References

[1] Feigin VL Brainin M Norrving B Martins S Sacco RL Hacke W Fisher M Pandian J Lindsay P. World Stroke Organization (WSO): global stroke fact sheet 2022. Int J Stroke. 2022;17:18–29. doi: 10.1177/17474930211065917 34986727 10.1177/17474930211065917

[2] Ekker MS Verhoeven JI Vaartjes I van Nieuwenhuizen KM Klijn CJM de Leeuw FE. Stroke incidence in young adults according to age, subtype, sex, and time trends. Neurology. 2019;92:e2444–e2454. Doi: 10.1212/WNL.0000000000007533 31019103 10.1212/WNL.0000000000007533

[3] Maaijwee NAMM Rutten-Jacobs LCA Arntz RM Schaapsmeerders P Schoonderwaldt HC van Dijk EJ de Leeuw FE. Long-term increased risk of unemployment after young stroke: a long-term follow-up study. Neurology. 2014;83:1132–1138. Doi: 10.1212/WNL.0000000000000817 25128177 10.1212/WNL.0000000000000817

[4] Kauranen T Turunen K Laari S Mustanoja S Baumann P Poutiainen E. The severity of cognitive deficits predicts return to work after a first-ever ischaemic stroke. J Neurol Neurosurg Psychiatry. 2013;84:316–321. Doi: 10.1136/jnnp-2012-302629 22952327 10.1136/jnnp-2012-302629

[5] Boehme AK Esenwa C Elkind MSV. Stroke risk factors, genetics, and prevention. Circ Res. 2017;120:472–495. Doi: 10.1161/CIRCRESAHA.116.308398 28154098 10.1161/CIRCRESAHA.116.308398 PMC5321635

[6] Strazzullo P D’Elia L Cairella G Garbagnati F Cappuccio FP Scalfi L. Excess body weight and incidence of stroke. Stroke. 2010;41:e418–e426. Doi: 10.1161/STROKEAHA.109.576967 20299666 10.1161/STROKEAHA.109.576967

[7] Mitchell AB Cole JW McArdle PF Cheng YC Ryan KA Sparks MJ Mitchell BD Kittner SJ. Obesity increases risk of ischemic stroke in young adults. Stroke. 2015;46:1690–1692. Doi: 10.1161/STROKEAHA.115.008940 25944320 10.1161/STROKEAHA.115.008940 PMC4458137

[8] Zou XL Wang S Wang LY Xiao LX Yao TX Zeng Y Zhang L. Childhood obesity and risk of stroke: a Mendelian dventist ion analysis. Front Genet. 2021;12:727475. Doi: 10.3389/fgene.2021.727475 34868204 10.3389/fgene.2021.727475 PMC8638161

[9] Simmonds M Llewellyn A Owen CG Woolacott N. Predicting adult obesity from childhood obesity: a systematic review and meta-analysis. Obes Rev. 2016;17:95–107. Doi: 10.1111/obr.12334 26696565 10.1111/obr.12334

[10] Jääskeläinen S Mäki P Peltomäki H Mäntymaa P. Lasten ja nuorten ylipaino ja lihavuus 2020 (Overweight and obesity in children and young 2020). Accessed September 3, 2022. https://www.julkari.fi/handle/10024/143273

[11] Finnish Institute for Health and Welfare. Obesity. 2022. Accessed September 3, 2022. https://thl.fi/fi/web/elintavat-ja-ravitsemus/lihavuus/lihavuuden-yleisyys

[12] Kivelä M Paananen M Kajantie E Ojaniemi M Nedelec R Rusanen H Miettunen J Rissanen I. Early childhood growth and risk of adult cerebrovascular disease: the NFBC1966. Stroke. 2022;53:1954–1963. Doi: 10.1161/STROKEAHA.121.035640 35300530 10.1161/STROKEAHA.121.035640

[13] Bushnell CD Chaturvedi S Gage KR Herson PS Hurn PD Jiménez MC Kittner SJ Madsen TE McCullough LD McDermott M. Sex differences in stroke: challenges and opportunities. J Cereb Blood Flow Metab. 2018;38:2179–2191. Doi: 10.1177/0271678X18793324 30114967 10.1177/0271678X18793324 PMC6282222

[14] University of Oulu: Northern Finland birth cohort 1966. University of Oulu. http://urn.fi/urn:nbn:fi:att:bc1e5408-980e-4a62-b899-43bec3755243

[15] Nordström T Miettunen J Auvinen J Ala-Mursula L Keinänen-Kiukaanniemi S Veijola J Järvelin MR Sebert S Männikkö M. Cohort profile: 46 years of follow-up of the Northern Finland Birth Cohort 1966 (NFBC1966). Int J Epidemiol. 2022;50:1786–1787j. doi: 10.1093/ije/dyab109 34999878 10.1093/ije/dyab109 PMC8743124

[16] World Health Organization. BMI-for-age boys. 2007 WHO reference. Accessed August 24, 2022. https://cdn.who.int/media/docs/default-source/child-growth/growth-reference-5-19-years/bmi-for-age-(5-19-years)/bmifa-boys-5-19years-per.pdf?sfvrsn=7ef4b722_4

[17] World Health Organization. BMI-for-age girls. 2007 WHO reference. Accessed August 24, 2022. https://cdn.who.int/media/docs/default-source/child-growth/growth-reference-5-19-years/bmi-for-age-(5-19-years)/bmifa-girls-5-19years-per.pdf?sfvrsn=b762eb2f_4

[18] Meschia JF Bushnell C Boden-Albala B Braun LT Bravata DM Chaturvedi S Creager MA Eckel RH Elkind MSV Fornage M; American Heart Association Stroke Council. Guidelines for the primary prevention of stroke. Stroke. 2014;45:3754–3832. Doi: 10.1161/STR.0000000000000046 25355838 10.1161/STR.0000000000000046 PMC5020564

[19] Tolonen H Salomaa V Torppa J Sivenius J Immonen-Räihä P Lehtonen A Finstroke R. The validation of the Finnish hospital discharge register and causes of death register data on stroke diagnoses. Eur J Cardiovasc Prev Rehabil. 2007;14:380–385. Doi: 10.1097/01.hjr.0000239466.26132.f2 17568236 10.1097/01.hjr.0000239466.26132.f2

[20] Rissanen I Oura P Paananen M Miettunen J Geerlings MI. Smoking trajectories and risk of stroke until age of 50 years – The Northern Finland birth cohort 1966. PloS One. 2019;14:e0225909. Doi: 10.1371/journal.pone.0225909 31846462 10.1371/journal.pone.0225909 PMC6917292

[21] Ohlsson C Bygdell M Sondén A Jern C Rosengren A Kindblom JM. BMI increase through puberty and adolescence is associated with risk of adult stroke. Neurology. 2017;89:363–369. Doi: 10.1212/WNL.0000000000004158 28659423 10.1212/WNL.0000000000004158 PMC5574671

[22] Rexrode KM Kimm SYS. Adolescent weight gain confers long-term increased stroke risk. Neurology. 2017;89:312–313. Doi: 10.1212/WNL.0000000000004160 28659422 10.1212/WNL.0000000000004160

[23] Prestgaard E Mariampillai J Engeseth K Erikssen J Bodegård J Liestøl K Kjeldsen S Grundvold I Berge E. Change in body weight and long-term risk of stroke and death in healthy men. Stroke. 2020;51:1435–1441. Doi: 10.1161/STROKEAHA.119.027233 32268850 10.1161/STROKEAHA.119.027233

[24] Osmond C Kajantie E Forsén TJ Eriksson JG Barker DJP. Infant growth and stroke in adult life: the Helsinki birth cohort study. Stroke. 2007;38:264–270. Doi: 10.1161/01.STR.0000254471.72186.03 17218608 10.1161/01.STR.0000254471.72186.03

[25] Quiñones-Ossa GA Lobo C Garcia-Ballestas E Florez WA Moscote-Salazar LR Agrawal A. Obesity and stroke: does the paradox apply for stroke? Neurointervention. 2021;16:9–19. Doi: 10.5469/neuroint.2020.00108 33389919 10.5469/neuroint.2020.00108 PMC7946563

[26] Ellulu MS Patimah I Khaza’ai H Rahmat A Abed Y. Obesity and inflammation: the linking mechanism and the complications. Arch Med Sci. 2017;4:851–863. Doi: 10.5114/aoms.2016.58928 10.5114/aoms.2016.58928 PMC5507106 28721154

[27] Shiozawa M Kaneko H Itoh H Morita K Okada A Matsuoka S Kiriyama H Kamon T Fujiu K Michihata N. Association of body mass index with ischemic and hemorrhagic stroke. Nutrients. 2021;13:2343. Doi: 10.3390/nu13072343 34371853 10.3390/nu13072343 PMC8308685

[28] Gu H Shao S Liu J Fan Z Chen Y Ni J Wang C Tu J Ning X Lou Y. Age- and sex-associated impacts of body mass index on stroke type risk: a 27-year prospective cohort study in a low-income population in China. Front Neurol. 2019;10:456. Doi: 10.3389/fneur.2019.00456 31118920 10.3389/fneur.2019.00456 PMC6504695

[29] Yonemoto K Doi Y Hata J Ninomiya T Fukuhara M Ikeda F Mukai N Iida M Kiyohara Y. Body mass index and stroke incidence in a Japanese community: the Hisayama study. Hypertens Res. 2011;34:274–279. Doi: 10.1038/hr.2010.220 21107333 10.1038/hr.2010.220

[30] Demel SL Kittner S Ley SH McDermott M Rexrode KM. Stroke risk factors unique to women. Stroke. 2018;49:518–523. Doi: 10.1161/STROKEAHA.117.018415 29438077 10.1161/STROKEAHA.117.018415 PMC5909714

[31] Holmegard HN Nordestgaard BG Jensen GB Tybjærg-Hansen A Benn M. Sex hormones and ischemic stroke: a prospective cohort study and meta-analyses. J Clin Endocrinol Metab. 2016;101:69–78. Doi: 10.1210/jc.2015-2687 26509870 10.1210/jc.2015-2687

[32] Canoy D Beral V Balkwill A Wright FL Kroll ME Reeves GK Green J Cairns BJ; Million Women Study Collaborators. Age at menarche and risks of coronary heart and other vascular diseases in a large UK cohort. Circulation. 2015;131:237–244. Doi: 10.1161/CIRCULATIONAHA.114.010070 25512444 10.1161/CIRCULATIONAHA.114.010070

[33] Jacobsen BK Oda K Knutsen SF Fraser GE. Age at menarche, total mortality and mortality from ischaemic heart disease and stroke: the dventist health study, 1976-88. Int J Epidemiol. 2009;38:245–252. Doi: 10.1093/ije/dyn251 19188208 10.1093/ije/dyn251 PMC2722816

[34] Cooper GS Ephross SA Weinberg CR Baird DD Whelan EA Sandler DP. Menstrual and reproductive risk factors for ischemic heart disease. Epidemiology. 1999;10:255–259. doi: 10.1097/00001648-199905000-00011 10230834

[35] Colditz G Willett W Stampfer M Rosner B Speizer F Hennekens C. A prospective study of age at menarche, parity, age at first birth, and coronary heart disease in women. Am J Epidemiol. 1987;126:861–870. Doi: 10.1093/oxfordjournals.aje.a114723 3661534 10.1093/oxfordjournals.aje.a114723

[36] Charalampopoulos D McLoughlin A Elks CE Ong KK. Age at menarche and risks of all-cause and cardiovascular death: a systematic review and meta-analysis. Am J Epidemiol. 2014;180:29–40. Doi: 10.1093/aje/kwu113 24920784 10.1093/aje/kwu113 PMC4070937

[37] Lazzeri G Tosti C Pammolli A Troiano G Vieno A Canale N Dalmasso P Lemma P Borraccino A Petraglia F. Overweight and lower age at menarche: evidence from the Italian HBSC cross-sectional survey. BMC Womens Health. 2018;18:168. Doi: 10.1186/s12905-018-0659-0 30340576 10.1186/s12905-018-0659-0 PMC6194651

[38] Antonopoulos AS Tousoulis D. The molecular mechanisms of obesity paradox. Cardiovasc Res. 2017;113:1074–1086. Doi: 10.1093/cvr/cvx106 28549096 10.1093/cvr/cvx106

[39] Engin A. The definition and prevalence of obesity and metabolic syndrome. Adv Exp Med Biol. 2017;960:1–17. Doi: 10.1007/978-3-319-48382-5_1 28585193 10.1007/978-3-319-48382-5_1

