# Supplementary material for: Overweight in Adolescence and Young Adulthood in Association With Adult Cerebrovascular Disease: The NFBC1966 Study
Source: Stroke. 2024 Jun 6;55(7):1857–65. doi: 10.1161/STROKEAHA.123.045444 (PMC11268552; doi:10.1161/STROKEAHA.123.045444)
Supplement: Supplementary file 1 [file str-55-1857-s001.pdf]

TABLE S1. Associations of BMI in adolescence and young and cerebrovascular event under the age of 55 years stratified by sex (without TIA).						
	**Any cerebrovascular disease**		**Ischemic cerebrovascular disease**		**Hemorrhagic cerebrovascular disease**	
**WOMEN**	N (%)	HR (95%CI)	N (%)	HR (95%CI)	N (%)	HR (95%CI)
**At 14 years**						
BMI (n=4788)						
16.00 – 22.99 (n=4213)	105 (2.5%)	ref	48 (1.1%)	ref	37 (0.9%)	ref
– 15.99 (n=217)	4 (1.8%)	0.77 (0.24-2.47)	1 (0.5%)	0.60 (0.08-4.40)	1 (0.5%)	N.A.
23.00 – 25.49 (n=247)	12 (4.9%)	**2.53 (1.34-4.78)**	6 (2.4%)	**2.53 (1.06-6.04)**	3 (1.3%)	1.55 (0.36-6.59)
25.50 – (n=111)	4 (3.6%)	1.96 (0.72-5.37)	2 (1.8%)	1.92 (0.46-7.96)	2 (1.9%)	3.08 (0.73-13.0)
**At 31 years**						
BMI (n=4276)						
20.00 – 24.99 (n=2424)	44 (1.8%)	ref	17 (0.7%)	ref	18 (0.8%)	ref
– 19.99 (n=593)	9 (1.5%)	1.10 (0.41-2.95)	5 (0.8%)	0.50 (0.06-3.96)	2 (0.3%)	0.99 (0.21-4.65)
25.00 – 29.99 (n=886)	29 (3.3%)	1.44 (0.66-3.12)	20 (2.3%)	2.55 (0.96-6.79)	3 (0.4%)	N.A.
30.00 – (n=373)	14 (3.8%)	**2.48 (1.07-5.72)**	4 (1.1%)	1.42 (0.30-6.70)	8 (2.2%)	**3.48 (1.13-10.7)**

**MEN**	N (%)	HR (95%CI)	N (%)	HR (95%CI)	N (%)	HR (95%CI)
**At 14 years**						
BMI (n=4820)						
16.00 – 21.99 (n=4046)	117 (2.9%)	ref	65 (1.6%)	ref	32 (0.8%)	ref
– 15.99 (n=224)	7 (3.1%)	1.15 (0.53-2.46)	4 (1.8%)	1.15 (0.42-3.15)	3 (1.3%)	1.84 (0.56-6.02)
22.00 – 24.29 (n=355)	14 (3.9%)	1.37 (0.78-2.38)	9 (2.5%)	1.57 (0.78-3.14)	2 (0.6%)	0.73 (0.17-3.04)
24.30 – (n=195)	9 (4.6%)	1.57 (0.80-3.09)	6 (3.1%)	1.86 (0.81-4.30)	3 (1.5%)	1.91 (0.58-6.26)
**At 31 years**						
BMI (n=4018)						
20.00 – 24.99 (n=1950)	54 (2.8%)	ref	31 (1.6%)	ref	12 (0.6%)	ref
– 19.99 (n=136)	2 (1.5%)	0.49 (0.07-3.57)	1 (0.7%)	N.A.	1 (0.7%)	3.20 (0.38-28.6)
25.00 – 29.99 (n=1585)	41 (2.6%)	1.10 (0.63-1.93)	21 (1.3%)	0.85 (0.40-1.82)	14 (0.9%)	2.19 (0.64-7.49)
30.00 – (n=347)	11 (3.2%)	1.08 (0.42-2.82)	6 (1.7%)	0.34 (0.05-2.55)	4 (1.2%)	**5.75 (1.43-23.1)**

Cox regression adjusted for smoking, parent's socioeconomic status, and the age at menarche for women at age 14; and for smoking pack years and one's own educational level at age 31. BMI=body mass index; HR=hazard ratio; N.A.=not applicable; and ref=reference group. BMI 16.00-22.99 or 16.00-21.99 or 20.00-24.99=normal weight; -15.99 or -19.99=underweight; 23.00-25.49 or 22.00-24.29 or 25.00-29.99=overweight; 25.50 or 24.30- or 30.00-=obesity.

TABLE S2. Interaction term of BMI and sex and cerebrovascular event under the age of 55 years in women compared to men (without TIA).

	Any cerebrovascular disease	Ischemic cerebrovascular disease	Hemorrhagic cerebrovascular disease
	RHR (95%CI)	RHR (95%CI)	RHR (95%CI)
**At 14 years**			
BMI (underweight) x Sex	0.66 (0.19-2.32)	0.37 (0.04-3.47)	0.23 (0.02-2.51)
BMI (overweight) x Sex	1.48 (0.66-3.37)	1.43 (0.47-4.30)	2.16 (0.32-14.2)
BMI (obese) x Sex	0.81 (0.24-2.73)	0.77 (0.15-3.98)	0.83 (0.12-5.53)
**At 31 years**			
BMI (underweight) x Sex	1.65 (0.17-16.4)	N.A.	0.23 (0.01-3.93)
BMI (overweight) x Sex	1.21 (0.45-3.27)	2.64 (0.70-9.99)	N.A.
BMI (obese) x Sex	2.09 (0.56-7.91)	N.A.	0.42 (0.06-2.81)

At age 14, the interaction model was adjusted with smoking, parent's socioeconomic status, and sex interactions of those. At age 31, the interaction model was adjusted with smoking, one's own socioeconomic status, and sex interactions of those.

TABLE S3. E-values of the results for BMI.

	**Any cerebrovascular disease**	**Ischemic cerebrovascular disease**	**Hemorrhagic cerebrovascular disease**
**WOMEN**	E-value for point estimate (for CI)	E-value for point estimate (for CI)	E-value for point estimate (for CI)
**At 14 years**			
BMI (n=4788)			
16.00 – 22.99 (n=4213)	ref	ref	ref
– 15.99 (n=217)	1.74 (1)	2.04 (1)	N.A.
23.00 – 25.49 (n=247)	4.42 (2.47)	4.42 (2.24)	2.47 (1)
25.50 – (n=111)	3.19 (1)	3.15 (1)	5.61 (1)
**At 31 years**			
BMI (n=4276)			
20.00 – 24.99 (n=2424)	ref	ref	ref
– 19.99 (n=593)	2.55 (1)	10 (1)	1.11 (1)
25.00 – 29.99 (n=886)	2.64 (1)	3.68 (1.54)	N.A.
30.00 – (n=373)	5.13 (2.47)	4.78 (1.83)	6.44 (1.51)

MEN	E-value for point estimate (for CI)	E-value for point estimate (for CI)	E-value for point estimate (for CI)
**At 14 years**			
BMI (n=4820)			
16.00 – 21.99 (n=4046)	ref	ref	ref
– 15.99 (n=224)	1.00 (1.00)	1.29 (1)	3.08 (1)
22.00 – 24.29 (n=355)	1.21 (1)	1.39 (1)	2.08 (1)
24.30 – (n=195)	1.69 (1)	1.69 (1)	3.23 (1)
**At 31 years**			
BMI (n=4018)			
20.00 – 24.99 (n=1950)	ref	ref	ref
– 19.99 (n=136)	5.33 (1)	N.A.	5.85 (1)
25.00 – 29.99 (n=1585)	1.88 (1)	1.69 (1)	3.8 (1)
30.00 – (n=347)	1.31 (1)	2.66 (1)	10.98 (2.21)

TABLE S4. E-values of the results for change in BMI among women.

At 14 years (adjusted for BMI at 31 years)	Any cerebrovascular disease	Ischemic cerebrovascular disease	Hemorrhagic cerebrovascular disease
	E-value for point estimate (for CI)	E-value for point estimate (for CI)	E-value for point estimate (for CI)
BMI (n=4788)			
16.00 – 22.99 (n=4213)	ref	ref	ref
– 15.99 (n=217)	2.21 (1)	2.81 (1)	N.A.
22.00 – 25.49 (n=247)	3.54 (1.79)	3.58 (1.59)	1.57 (1)
25.50 – (n=111)	2.39 (1)	2.54 (1)	2.32 (1)
**At 31 years (adjusted for BMI at 14 years)**	**Any cerebrovascular disease**	**Ischemic cerebrovascular disease**	**Hemorrhagic cerebrovascular disease**
	E-value for point estimate (for CI)	E-value for point estimate (for CI)	E-value for point estimate (for CI)
BMI (n=4276)			
20.00 – 24.99 (n=2424)	ref	ref	ref
– 19.99 (n=593)	1.85 (1)	7.8 (1)	1.62 (1)
25.00 – 29.99 (n=886)	3.1 (1)	4.21 (1.67)	N.A.
30.00 – (n=373)	5.51 (2.28)	5.31 (1.69)	5.87 (1)

TABLE S5. E-values of the results for change in BMI among men.

At 14 years (adjusted for BMI at 31 years)	Any cerebrovascular disease	Ischemic cerebrovascular disease	Hemorrhagic cerebrovascular disease
	E-value for point estimate (for CI)	E-value for point estimate (for CI)	E-value for point estimate (for CI)
BMI (n=4820)			
16.00 – 21.99 (n=4046)	ref	ref	ref
– 15.99 (n=224)	1.36 (1)	1.63 (1)	3.43 (1)
22.00 – 24.29 (n=355)	1.6 (1)	1.46 (1)	2.26 (1)
24.30 – (n=195)	1.5 (1)	1.88 (1)	1.43 (1)
**At 31 years (adjusted for BMI at 14 years)**	**Any cerebrovascular disease**	**Ischemic cerebrovascular disease**	**Hemorrhagic cerebrovascular disease**
	E-value for point estimate (for CI)	E-value for point estimate (for CI)	E-value for point estimate (for CI)
BMI (n=4018)			
20.00 – 24.99 (n=1950)	ref	ref	ref
– 19.99 (n=136)	4.31 (1)	N.A.	9.43 (1)
25.00 – 29.99 (n=1585)	2.15 (1)	2.06 (1)	8.07 (1)
30.00 – (n=347)	2.04 (1)	2.9 (1)	38.29 (6.01)

TABLE S6. E-values for the interaction terms compared to men.

	Any cerebrovascular disease	Ischemic cerebrovascular disease	Hemorrhagic cerebrovascular disease
	E-value for point estimate (for CI)	E-value for point estimate (for CI)	E-value for point estimate (for CI)
**At 14 years**			
BMI (underweight) x Sex	1.11 (1)	1.46 (1)	8.16 (1)
BMI (overweight) x Sex	3.6 (1.31)	4.01 (1.11)	3.74 (1)
BMI (obese) x Sex	1.86 (1)	2.26 (1)	1.7 (1)
**At 31 years**			
BMI (underweight) x Sex	2.58 (1)	N.A.	8.16 (1)
BMI (overweight) x Sex	1.74 (1)	2.92 (1)	N.A.
BMI (obese) x Sex	5.13 (1)	13.4 (2.06)	4.19 (1)

TABLE S7. E-values for the results for BMI (without TIA).			
	**Any cerebrovascular disease**	**Ischemic cerebrovascular disease**	**Hemorrhagic cerebrovascular disease**
**WOMEN**	E-value for point estimate (for CI)	E-value for point estimate (for CI)	E-value for point estimate (for CI)
**At 14 years**			
BMI (n=4788)			
16.00 – 22.99 (n=4213)	ref	ref	ref
– 15.99 (n=217)	1.92 (1)	2.72 (1)	N.A.
23.00 – 25.49 (n=247)	4.5 (2.01)	4.5 (1.31)	2.47 (1)
25.50 – (n=111)	3.33 (1)	3.25 (1)	5.61 (1)
**At 31 years**			
BMI (n=4276)			
20.00 – 24.99 (n=2424)	ref	ref	ref
– 19.99 (n=593)	1.43 (1)	3.41 (1)	1.11 (1)
25.00 – 29.99 (n=886)	2.24 (1)	4.54 (1)	N.A.
30.00 – (n=373)	4.4 (1.34)	2.19 (1)	6.42 (1.51)

**MEN**	E-value for point estimate (for CI)	E-value for point estimate (for CI)	E-value for point estimate (for CI)
**At 14 years**			
BMI (n=4820)			
16.00 – 21.99 (n=4046)	ref	ref	ref
– 15.99 (n=224)	1.57 (1)	1.57 (1)	3.08 (1)
22.00 – 24.29 (n=355)	2.08 (1)	2.52 (1)	2.08 (1)
24.30 – (n=195)	2.52 (1)	3.12 (1)	3.23 (1)
**At 31 years**			
BMI (n=4018)			
20.00 – 24.99 (n=1950)	ref	ref	ref
– 19.99 (n=136)	2.34 (1)	N.A.	5.85 (1)
25.00 – 29.99 (n=1585)	1.43 (1)	1.63 (1)	3.8 (1)
30.00 – (n=347)	1.37 (1)	5.33 (1)	10.98 (2.21)

TABLE S8. E-values for the interaction terms compared to men (without TIA).

	Any cerebrovascular disease	Ischemic cerebrovascular disease	Hemorrhagic cerebrovascular disease
	E-value for point estimate (for CI)	E-value for point estimate (for CI)	E-value for point estimate (for CI)
**At 14 years**			
BMI (underweight) x Sex	2.4 (1)	4.85 (1)	8.16 (1)
BMI (overweight) x Sex	2.32 (1)	2.21 (1)	3.74 (1)
BMI (obese) x Sex	1.77 (1)	1.92 (1)	1.7 (1)
**At 31 years**			
BMI (underweight) x Sex	2.69 (1)	N.A.	8.16 (1)
BMI (overweight) x Sex	1.71 (1)	4.72 (1)	N.A.
BMI (obese) x Sex	3.6 (1)	N.A.	4.19 (1)
